# Rethinking Outreach: Teaching the Process of Science through Modeling

**DOI:** 10.1371/journal.pbio.0060086

**Published:** 2008-04-08

**Authors:** Tim Herman, Shannon Colton, Margaret Franzen

## Abstract

How can we get high school students interested in science? Here is a program that matches students with researchers, with the purpose of building a physical model of the protein being investigated in the lab.

*“Oh,…you mean if you want to change the amino acid from an arginine to an alanine, you just change the code in that position.*” This “aha moment” recently happened for a member of the Brown Deer High School SMART (Students Modeling a Research Topic) Team at a Saturday morning meeting while awaiting the arrival of their scientist mentor. This student had just realized that the process used by scientists to create modified proteins, and to test the impact of that modification on function, involves engineering the gene for the protein, and then expressing the modified gene in an appropriate system. The excitement of this new insight spread throughout the team as other students picked up this new information and began to reinterpret some elements of “the story” that had been told to them by their scientist mentor at a previous meeting. Their mentor's story about the tumor suppressor maspin and its role in metastasis was now becoming “their story”—as they began to truly understand not only what the maspin protein was and how it functioned, but also the process of science whereby their mentor had designed an experiment that resulted in the data that supported the story. And the story was becoming all the more compelling because the students had in hand a physical model of the maspin protein they had designed and built using rapid prototyping technology (see Figure 1).

**Figure 1:**
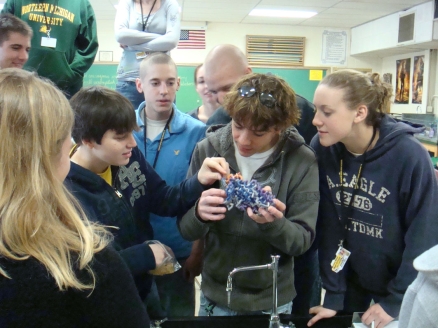
Creating the “Aha” Moment Members of the Brown Deer High School SMART Team examine the alanine-to-arginine mutation on their physical model of the maspin protein.

There has been a recent call for undergraduate faculty to engage in “scientific teaching”—a way of active teaching that places equal emphasis on both the process of science and the facts of science [1]. We endorse this approach and believe that the Milwaukee School of Engineering (MSOE) SMART Team modeling program captures many of the principles of scientific teaching in a high school outreach program by exposing students to the “real world of science” as practiced in a local research lab. SMART Teams, which consist of a small group of high school students and their teacher, work with a local research lab to design and build a physical model of the protein that is the focus of the lab's research. During this modeling project, the students learn that science is much more than just the facts documented in their textbooks. They see that science is a process whereby real people—undergraduates, graduate students, post-docs, and principal investigators—go about learning something about a molecular world invisible to the naked eye. And in the molecular biosciences, what they are learning can be pretty amazing. While experiencing the culture of a research lab, SMART Team students begin to imagine themselves in the roles of their scientist mentors.

The SMART Team program grew out of a professional development summer course for high school science teachers offered by the MSOE Center for BioMolecular Modeling. In this course, “Genes, Schemes, and Molecular Machines,” teachers explore how current concepts of molecular structure and function can be introduced to students as natural extensions of basic principles of chemistry and physics. In addition, teachers learn how physical models of proteins in the classroom can serve as compelling props that capture the interest of students and stir a desire to learn more. The physical models are based on atomic coordinates and are designed using RP-RasMol (http://www.rpc.msoe.edu/cbm/about/technology.php), a modified version of RasMol, molecular visualization software that allows users to examine protein structures in a computer environment. Any image of a protein that can be created in RP-RasMol can be exported as a file that can be recognized by rapid prototyping equipment, and then built in an automated, layer-by-layer process using a variety of materials including plaster, nylon, paper, or a liquid photopolymer. After participating in this course, high school teachers, and then their students, become protein model designers and builders.

SMART Teams were born in the fall of 2001 when Jeff Anderson, a high school teacher at Riverside University High School in Milwaukee, approached us with an idea for a student project. Jeff had participated in the “Genes and Schemes” summer course, and had heard about a group of his fellow teachers who had just designed and built the first-ever physical models of the ribosome, based on atomic coordinate data published by the Steitz and Noller laboratories [2,3]. Jeff wanted to get his students involved in a similar modeling project and decided to focus on anthrax, which received substantial press coverage after anthrax-laced letters had been sent first to news outlets and later to two US senators. The three high school students on this first SMART Team began by modeling the three proteins involved in anthrax pathogenesis—edema factor, protective antigen heptamer, and lethal factor.

During this project, the students met with two researchers who were actively involved in this work, Wei-Jen Tang of the University of Chicago, and John Young, then at the University of Wisconsin–Madison (see Figure 2). Just as timing plays a big role in successful science breakthroughs, timing was critical to the success of this first SMART Team project. Tang had just solved the structure of the anthrax edema factor when he was contacted by the SMART Team, who offered to work with him to design and build a physical model of the protein. As a result, when the press arrived at Tang's lab in February of 2002 to interview him on the day his paper was published in *Nature* [4], he had a full collection of physical models of all three anthrax proteins to use as props as he discussed his work. Several months later, when Young and John Collier traveled to Washington to address a special congressional subcommittee on measures to counter the threat of bioterrorism, they were able to distribute 24 copies of the SMART Team's anthrax protective antigen heptamer model to members of the committee during the testimony.

**Figure 2:**
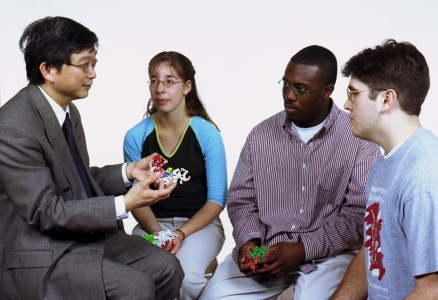
Structural Insights into Anthrax Pathogenesis Wei-Jen Tang (left) discusses a physical model of the anthrax edema factor with the Riverside High School SMART Team.

Since the auspicious beginning of that first fortuitous SMART Team, the program has grown to 14 teams that annually work with researchers in southeastern Wisconsin. The current size of the program is limited only by the capacity of our outreach program to coordinate the teams' activities. Local research labs interested in hosting SMART Teams far exceed the number of teams. The popularity of the program among research labs is a testament to its minimally invasive nature. In contrast to many traditional outreach programs that involve immersive research experiences for small numbers of high school students or teachers, a SMART Team program, recognizing the current pressure felt by researchers to remain productive and maintain research funding, minimizes the formal interactions between the team and lab to only three to five meetings over the course of the project. In addition, the interaction represents a true partnership that occurs on a relatively level playing field in which both parties benefit. The students and their teacher benefit from exposure to the culture of a research lab and the details of a specific research project. The research lab benefits from the refreshing challenge of discussing their project with a novice audience, against a broad background of answering that fundamental question, “Why is this important?” As an added bonus, the research lab receives a copy of the physical model of the protein they are investigating. This physical model becomes a valuable communication tool within the lab.

The development of the SMART Team program has been supported by a Science Education Partnership Award to the MSOE Center for BioMolecular Modeling from the National Center for Research Resources of the National Institutes of Health. In addition to our local program in Wisconsin, approximately 14 additional Remote SMART Teams operate independently each year in high schools from New Jersey to California. More recently, a precollege science education award from the Howard Hughes Medical Institute has funded a project to disseminate this SMART Team program at eight universities across the US, including Rockefeller, Stony Brook, Rutgers, West Virginia University, Washington University, the University of Wisconsin–Madison, Scripps, the University of California, San Diego, the University of California San Francisco, and the Fred Hutchinson Cancer Research Center. Anyone interested in participating in a SMART Team program, either as a scientist mentor or an outreach staff member interested in starting a program at their institution, should contact us via our Web site at http://www.rpc.msoe.edu/cbm.

## Abbreviations

SMART: Students Modeling a Research Topic
MSOE: Milwaukee School of Engineering
